# Tong-Xie-Yao-Fang improves intestinal permeability in diarrhoea-predominant irritable bowel syndrome rats by inhibiting the NF-κB and notch signalling pathways

**DOI:** 10.1186/s12906-019-2749-4

**Published:** 2019-11-27

**Authors:** Qiuke Hou, Yongquan Huang, Zhaoyang Zhu, Liu Liao, Xinlin Chen, Quanbin Han, Fengbin Liu

**Affiliations:** 1 Department of Gastroenterology, The First Affiliated Hospital of Guangzhou University of Chinese Medicine, Guangzhou, 510405 Guangdong China; 2 School of Chinese Medicine, Hong Kong Baptist University, Hong Kong, China; 3 Department of Orthopaedics, The Second Affiliated Hospital of Guangzhou University of Chinese Medicine, Guangzhou, China; 4 Department of Preventive Medicine and Health Statistics, Guangzhou University of Chinese Medicine, Guangzhou, Guangdong China

**Keywords:** Tong-Xie-Yao-fang, Diarrhoea predominant-irritable bowel syndrome, Intestinal permeability, NF-κB Signalling, Notch Signalling

## Abstract

**Background:**

Tong-Xie-Yao-Fang (TXYF) has been shown to be effective in diarrhoea-predominant irritable bowel syndrome (IBS-D) patients. However, the underlying mechanism remains to be clarified. The aim of this study was to investigate the efficacy and related mechanisms of TXYF in an IBS-D rat model.

**Methods:**

The IBS-D rat model was established with 4% acetic acid and evaluated by haematoxylin-eosin (HE) staining. Then, IBS-D rats were divided into control, TXYF and rifaximin groups and treated intragastrically with normal saline, TXYF and rifaximin, respectively, for 14 days. The following indicators were measured before and after treatment: defecation frequency, faecal water content (FWC) and colorectal distension (CRD). Histopathological changes in the distal colon were observed after treatment. The expression of OCLN and ZO1 in the distal colon of IBS-D rats reflected the intestinal mucosal permeability, as measured by qRT-PCR, western blot, and enzyme-linked immunosorbent assays (ELISAs). The NF-κB and Notch signalling pathways and inflammation-related factors were investigated.

**Results:**

After treatment with TXYF, the defecation frequency, FWC and CRD were significantly lower than those in the model group (*P* < 0.05). HE staining showed that colonic epithelial cells (CECs) in the IBS-D rats displayed significant oedema, impaired intestinal mucosal integrity and an increased influx of inflammatory cells. A significant reduction in granulocyte and CEC oedema was observed after the administration of TXYF and rifaximin compared to that of the model group and blank group (*P* < 0.05). TXYF significantly upregulated the expression of OCLN and ZO-1 and downregulated inflammation-related factors (IL-6, IL-1β, and TNF-α and the chemokine KC) in IBS-D rats compared to those in the model group rats (*P* < 0.05). In terms of the NF-κB and Notch signalling pathways, the expression of NICD, p-ERK, Hes-1 and p-P65 decreased significantly in the TXYF and rifaximin groups, while the expression of ATOH1 increased significantly compared to that in the model group (*P* < 0.05).

**Conclusion:**

TXYF can effectively improve intestinal permeability and enhance intestinal mucosal barrier function, which may be related to inhibition of the inflammatory cascade and the NF-κB and Notch signalling pathways.

## Background

Diarrhoea-predominant irritable bowel syndrome (IBS-D) is generally reported as the most common subtype (28–46%) of IBS [1], which is characterized by altered habits of bowel evacuation, visceral pain, and bloating in the absence of anatomical or biochemical abnormalities. In the past, most reports focused on gastrointestinal motility disorders or visceral hypersensitivity of the distal colon [2, 3]. In addition, our previous study showed that IBS-D rats have an increased degree of intestinal permeability [4].

According to our previous data, miR-144 regulates intestinal permeability during IBS-D as an effective modulator of the intestinal tight junction proteins [4]. It has also been reported that IBS-D is caused by stress-induced brain-intestinal axis changes, leading to excessive intestinal permeability, which aggravates submucosal abnormal immune responses [5, 6]. However, the exact pathophysiological signals leading to impaired intestinal mucosal barrier function in IBS-D are still unclear.

Tong-Xie-Yao-Fang (TXYF) is one of the classic prescriptions of traditional Chinese medicine. It consists of four crude drugs: *Rhizoma Atractylodis Macrocephalae*, *Radix Paeoniae Alba*, *Pericarpium Citri Reticulatae* and *Radix Saposhnikoviae*. This prescription has been widely used for clinical treatment of IBS-D in China for nearly 600 years, with reliable efficacy, few side effects and a low recurrence rate [7, 8], which is especially suitable for IBS-D patients with disharmony of the liver and spleen. There have been many reports in the literature on the treatment of the IBS-D animal model by Chinese herbal compounds to explore its pharmacodynamics and mechanism [9–12] and reports on the effect of TXYF on the IBS-D animal model [13, 14]. However, the mechanism of TXYF in relieving abnormal immune responses and regulating intestinal permeability remains unclear.

In this study, we aimed to explore the underlying mechanism of TXYF treatment in IBS-D rats and discuss the pathophysiological signals leading to the destruction of intestinal mucosal barrier function from two aspects: intestinal permeability and immune regulation.

## Methods

### Materials

Acetic acid was purchased from Hamilton Cells (Wuhan, China). Antibodies against p65, P-p65, NICD, p-ERK, ERK, OCLN and ZO-1 were purchased from Cell Signaling Technology (Danvers, Colorado, USA), and a β-actin antibody was obtained from ABclonal Biotech Co., Ltd. (Wuhan, China). HRP-conjugated goat anti-rabbit/mouse IgG was purchased from Vazyme Biotech Co., Ltd. (Nanjing, China). ELISA kits for rat OCLN and ZO-1 were purchased from R&D Systems China Co., Ltd. (Shanghai, China). RT-PCR primers were synthesized by Invitrogen (Shanghai, China). Primer synthesis was performed by Takara BioInc (Takara BioInc, Dalian, China). TRIzol and cDNA synthesis kits were obtained from Invitrogen (Carlsbad, CA, USA). All other chemicals were purchased from Sigma-Aldrich.

### Preparation of Tong-Xie-Yao-Fang

TXYF consists of four crude drugs: *Rhizoma Atractylodis Macrocephalae* (18 g), *Radix Paeoniae Alba* (12 g), *Pericarpium Citri Reticulatae* (9 g) and *Radix Saposhnikoviae* (6 g) (Table 1), corresponding to the common dose for adult humans, which was obtained from Kangmei Pharmacy (Guangzhou, China). Then, the four crude drugs were extracted and dried to acquire powder of TXYF’s aqueous extract, which was made by the Institute of Translational Medicine of Guangzhou University of Traditional Chinese Medicine. In brief, 45 g of crude TXYF was placed in 600 mL of purified water for 12 h, boiled for 2 h, and then filtered to acquire the liquid. The extract was dried in a vacuum drying oven until no liquid was present; then, 15 g of powder of TXYF’s aqueous extract was obtained, which indicated that each gram of powder contained 3 g of the initial crude drugs.

**Table 1:** Ingredients of of TXYF^a^

Ingredients (Latin name)	Quantity (dry, g)
*Rhizoma Atractylodis Macrocephalae*	18
*Radix Paeoniae Alba*	12
*Pericarpium Citri Reticulatae*	9
*Radix Saposhnikoviae*	6

^a^common dose for adult humans

### IBS-D rat model construction and treatment

Forty (male or female) specific pathogen-free Sprague-Dawley rats (4 weeks old, weighing 140 ± 10 g) were obtained from the Experimental Animal Center of Guangzhou University of Chinese Medicine (Guangzhou, China) and then randomly averaged divided into four groups: a blank group (10), model group (10), TXYF group (10) and rifaximin group (10). Rats were housed at the Experimental Animal Center of Guangzhou University of Chinese Medicine at 22 ± 1 °C and 50 ± 70% humidity under a 12-h light-dark cycle. Rats were provided free access to food and water in their cage. The IBS-D rat model was performed as we previously reported [4]. In brief, the model group, TXYF group, and rifaximin group rats were intracolonically instilled with 1 mL of 4% acetic acid at 8 cm proximal to the anus for 30 s after being lightly anaesthetized with ether (inhalation, 30 s). Then, 1 mL of phosphate-buffered saline (PBS) was instilled to dilute the acetic acid and flush the colon. The blank group was handled identically to the other groups, except that 1 mL of PBS was instilled instead of 4% acetic acid. The rats were allowed to recover for 6 days. On the 7th day, 5 rats per group were randomly selected for model evaluation of defecation frequency, faecal water content (FWC) and colorectal distension (CRD); then, they were sacrificed by cervical dislocation to obtain the distal colon, which was stained with haematoxylin-eosin (HE) to explore the pathological change due to IBS-D.

The remaining 20 rats (5 in each group) were treated as follows. The blank group was treated with normal saline [10 mL/kg, intragastric administration (ig)]. The rifaximin group was treated with rifaximin suspended in liquid (13.5 × 10^− 3^ g/kg, ig). The TXYF group was treated with a TXYF suspension (4.725 g/kg, ig). Each group was dosed 2 times per day for a total of 2 weeks. On the 15th day, the defecation frequency, FWC and AWR score of all rats were measured again, and then the rats were sacrificed by cervical dislocation to obtain the distal colon for future experiments, such as HE staining, western blot, qRT-PCR and enzyme-linked immunosorbent assays (ELISA).

### Defecation frequency and FWC

Defecation frequency and FWC were used to estimate colonic motility and permeability. The rats were placed in cages for 24 h with free access to food and water. The faecal pellets were counted and weighed (m_0_) after collection, the average number of each hour count was calculated as the defecation frequency, and the faecal pellets were weighed again (m_1_) after they were dried in the oven. FWC was calculated as (m_0_ – m_1_)/m_0_.

### CRD

Abdominal withdrawal reflex (AWR) scores were used to quantify the CRD, which has been described previously [14]. Simply, the rats were placed in a small cubicle and allowed to adapt for 15 min. Then, a balloon dilator (2 mm in diameter) was Vaseline coated and inserted into the descending colon, which was located 8 cm proximal to the anus. Then, water was injected into the balloon, leading to CRD. The AWR score was measured by two independent observers using a double-blind method based on the following criteria: 0 points: no behavioural response to CRD; 1 point: simple head movement, then no movement; 2 points: abdominal muscle contraction; 3 points: abdominal rise; and 4 points: back arch and ascending pelvis. When the AWR score was 3, the amount of injected water was calculated as the degree of CRD. The experiment was repeated in triplicate to obtain an average value.

### HE staining

After the rats were sacrificed, the distal colon was harvested, rinsed and fixed in 4% paraformaldehyde overnight at 4 °C. Colonic tissues were then treated with a continuous cleaning and dehydration step and embedded in a paraffin block. Next, the colonic tissues were sliced and subjected to standard HE staining for the evaluation of intestinal mucosal barrier integrity of mucosal layers and the influx of inflammatory cells. Three samples were selected from each group, and four regions were selected from each sample, which were observed blindly by three independent observers.

### ELISAs

We measured the levels of OCLN and ZO1 in colonic tissues. Colonic tissues were digested and homogenized in 0.25% trypsin and then centrifuged for 30 mins (3000 rpm). The supernatant was collected. The content of OCLN and ZO1 in the supernatant was determined using a suitable ELISA kit (R&D Systems, Minneapolis, MN, USA) according to the manufacturer’s instructions at 1 day before treatment and after 2 weeks of treatment. Then, the optical density (OD) value was measured at 450 nm using a microplate reader to determine the concentrations of OCLN and ZO1. The experiment was repeated in triplicate to obtain an average OD value.

### Western blot analysis

Western blot analysis followed the standard procedure we previously reported [4]. In brief, colonic tissues were homogenized in ice-cold RIPA buffer containing a protease inhibitor. Ten micrograms of the protein sample was separated by SDS-PAGE and electrophoretically transferred to a polyvinylidene fluoride membrane (Bio-Rad). After blocking with 5% skim milk, the membrane was incubated with the primary antibody and then incubated with the appropriate secondary antibody conjugated to HRP. Positive western blots were detected with X-ray film (Fuji) by chemiluminescence using an ECL kit (GE Healthcare).

### Total RNA extraction and quantitative real-time PCR (qRT-PCR)

*qRT-PCR* analysis followed the standard procedure we previously reported [4]. In brief, total RNA was extracted using a total RNA extraction kit (Omega, Norcross, GA, USA). A RevertAid First Strand cDNA kit (Thermo Fisher Scientific, Waltham, MA, USA) was used to synthesize complementary DNA (cDNA). qRT-PCR was performed on an ABI Prism 7500 PCR system (Applied Biosystems, USA) using a Platinum SYBR Green qRT-PCR SuperMix-UDG kit (Life Technologies). β-actin was used as an internal reference, and the relative expression level of the target protein was calculated using the 2^-ΔΔCt^ method.

### Statistical analysis

All statistical analyses were performed using SPSS version 19 (Stanford, CA, USA). Data were expressed as the means±standard deviations (SDs) and analysed by one-way ANOVA followed by Tukey’s multiple comparison test to detect inter-group differences. Significant differences from the control group were determined at 95% confidence intervals. The threshold of statistical significance was set to *P* < 0.05.

## Results

### TXYF ameliorated symptoms in IBS-D rats

**Fig. 1:**
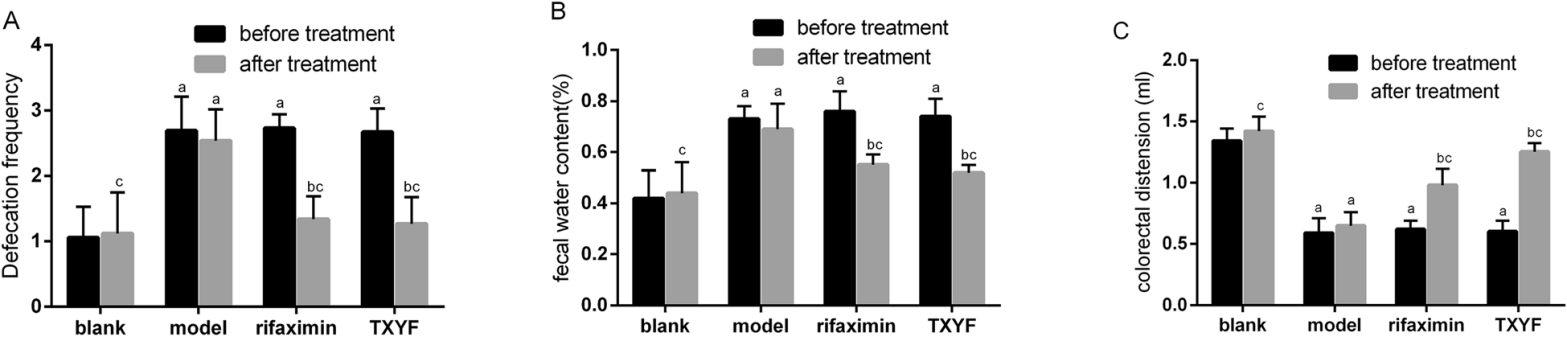
Effects of TXYF on defecation frequency, FWC and CRD in IBS-D rats. **a** Defecation frequency, (**b**) faecal water content, and (**c**) colorectal distension. ^a^*P* < 0.05 vs the blank group. ^b^*P* < 0.05 vs before treatment. ^c^*P* < 0.05 vs the model group

Defecation frequency and FWC were significantly increased, while CRD was significantly decreased in IBS-D rats when compared to those in the blank group rats. After treatment for 14 days, the defecation frequency and FWC were significantly decreased when compared to those in the model group (*P* < 0.05), while there was no difference between the rifaximin and TXYF groups (Fig. 1a & b). The CRD in the TXYF group and rifaximin group were remarkably increased when compared to those in the model group (*P* < 0.05), and those of the TXYF group increased the most (Fig. 1c). These results strongly supported the successful establishment of the IBS-D rat model and that TXYF significantly ameliorated symptoms in IBS-D rats.

### TXYF promotes histological recovery in IBS-D rats

**Fig. 2:**
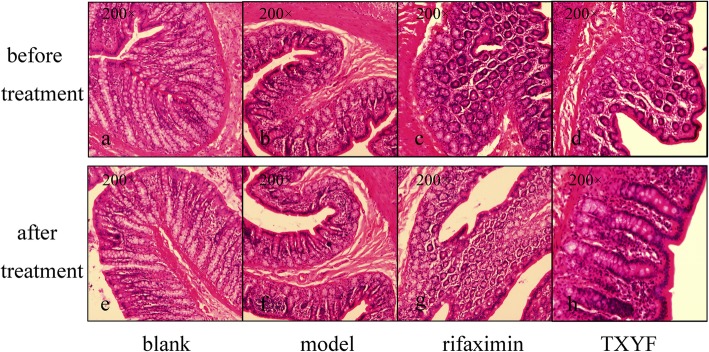
Histological examination of the intestinal mucosal barrier. **a**-**d** One day before treatment; **e**-**h** after 2 weeks of treatment

The intestinal mucosal barrier in the normal rats (blank group) showed integrity mediated by colonic epithelial cells (CECs), whereas CECs in the IBS-D rats displayed significant oedema, impaired intestinal mucosal integrity and an increased influx of inflammatory cells (Fig. 2a-d). Histological analysis of rat colonic tissues revealed a significant reduction in granulocyte and CEC oedema after the administration of TXYF and rifaximin compared to those in the tissues of the model group and blank group (Fig. 2e-f). The intestinal mucosal barrier integrity of mucosal layers was repaired after oral administration of TXYF and rifaximin, and there was no significant difference between the TXYF and rifaximin groups (Fig. 2g, h). This result strongly suggested that TXYF could significantly decrease intestinal permeability and promote histological recovery in IBS-D rats.

### TXYF upregulated the expression of OCLN and ZO1 in IBS-D rats

**Fig. 3:**
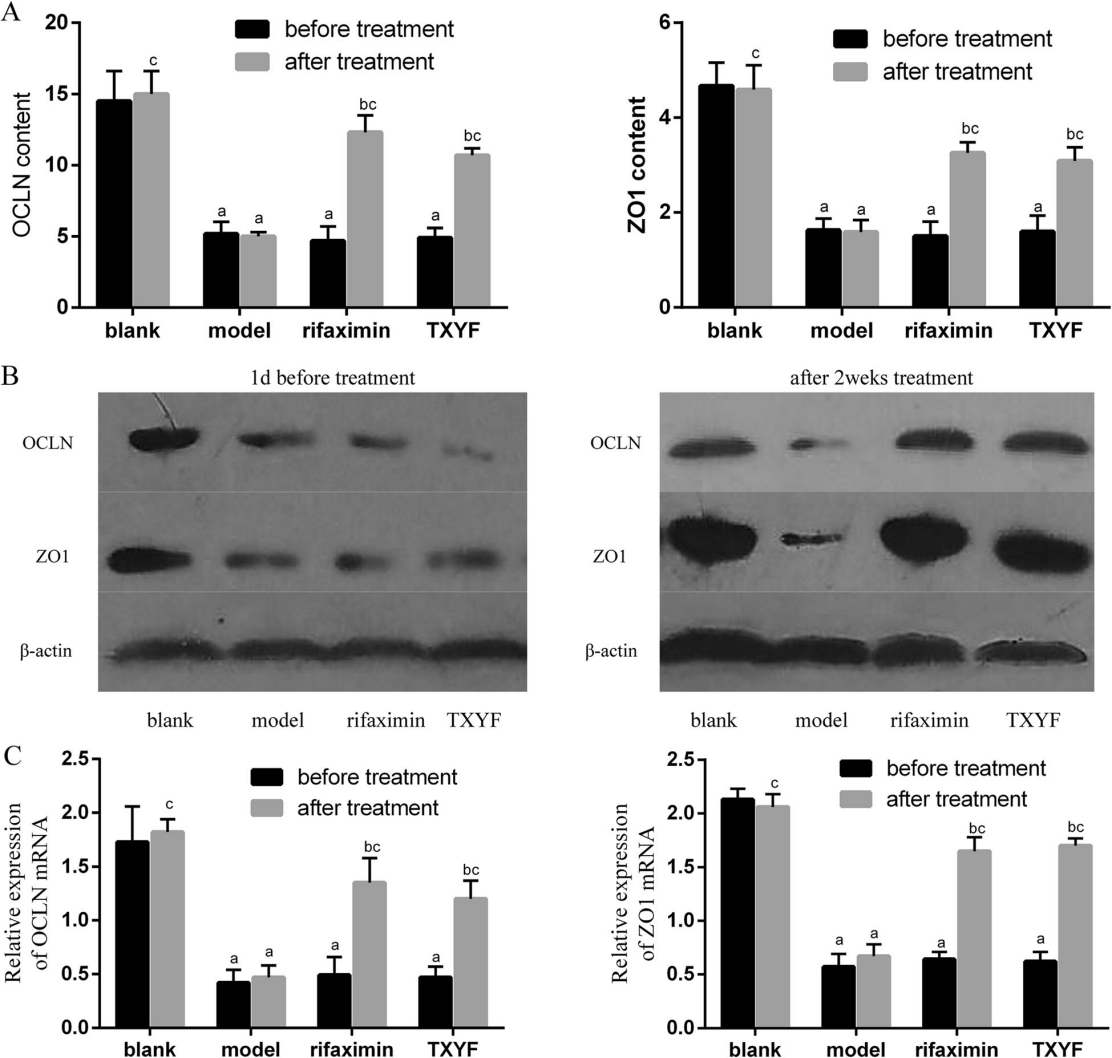
TXYF protects epithelial permeability in the colon of IBS-D rats. **a** ELISAs showed that the OCLN and ZO1 contents were significantly decreased in the model, rifaximin and TXYF groups before treatment but increased in the rifaximin and TXYF groups after treatment. **b** Western blot analysis for OCLN and ZO1 in colonic tissue samples. **c** qRT-PCR for OCLN and ZO1 in colonic tissue samples. ^a^*P* < 0.05 vs the blank group. ^b^*P* < 0.05 vs before treatment. ^c^*P* < 0.05 vs the model group

Since OCLN and ZO1 are the most important transmembrane proteins that impact the permeability of tight junctions in colonic tissues, their expression in colonic tissues was investigated by ELISA, western blot and qRT-PCR. As shown in Fig. 3a, ELISA revealed that the content of OCLN and ZO1 was downregulated in the IBS-D model group compared with that in the blank group, suggesting that intestinal mucosal barrier function was impaired in IBS-D rats. The level of OCLN and ZO1 was upregulated after treatment when compared to that of the model group. However, no differences were found between the TXYF and rifaximin groups. We also examined the expression levels of OCLN and ZO1 by western blot and qRT-PCR (Fig. 3b and c), and the results were in accordance with the ELISA results, which strongly suggested that TXYF and rifaximin are involved in improving intestinal permeability in IBS-D rats. The above results strongly suggested that TXYF could significantly enhance intestinal mucosal barrier function in IBS-D rats.

### TXYF inhibits the notch signalling pathway in IBS-D rats

**Fig. 4:**
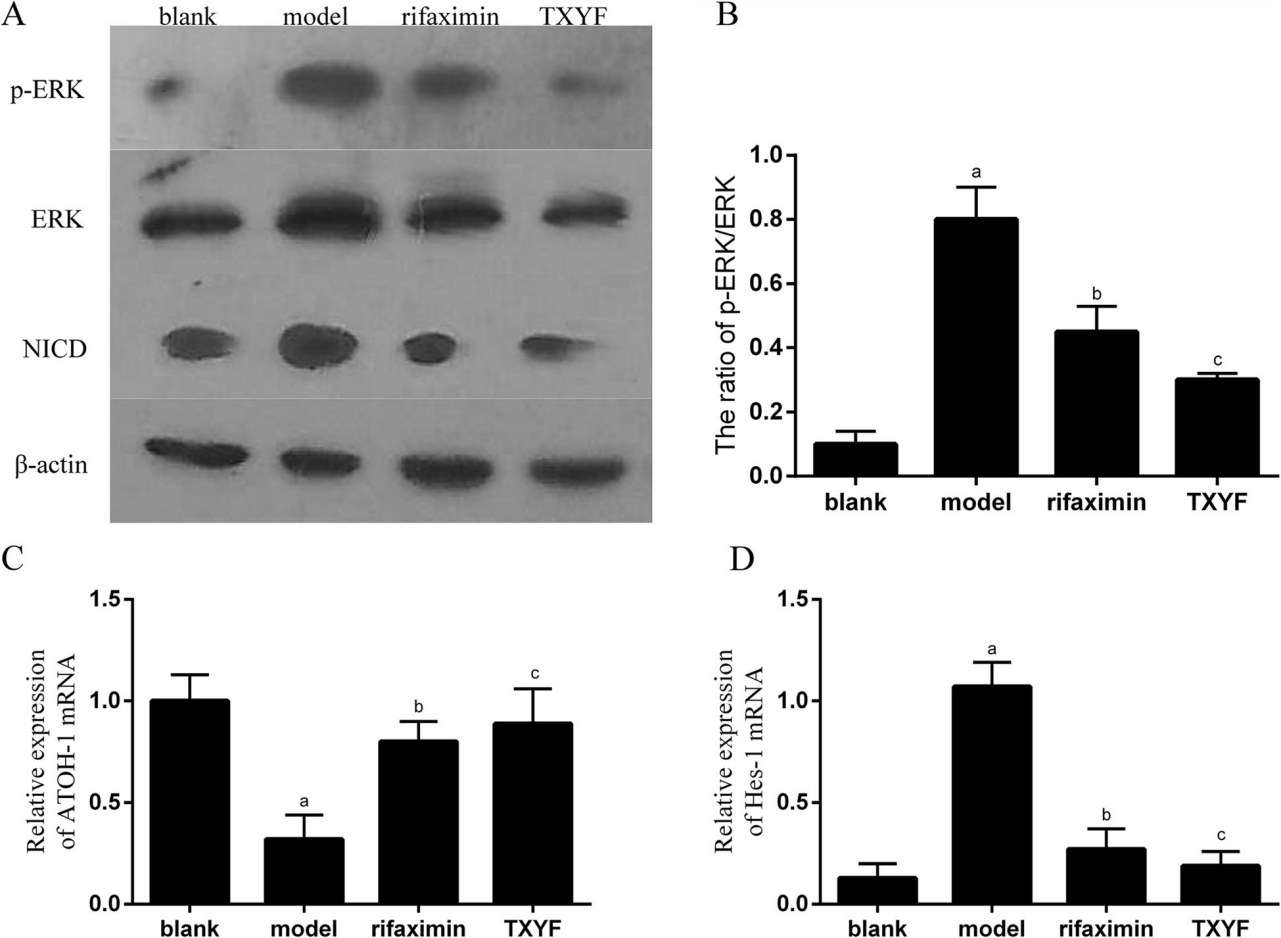
TXYF inhibits the Notch signalling pathway in IBS-D rats. **a** Western blot analysis for the expression of NICD, p-ERK and ERK in distal colonic tissues. **b** The ratio of p-ERK/ERK. **c**, **d** qRT-PCR detection of Hes-1 and ATOH1 in distal colonic tissues. ^a^*P* < 0.05 vs the blank group. ^b^*P* < 0.05 vs the model group. ^c^*P* > 0.05 vs the rifaximin group

Because the Notch signalling pathway plays a key role in the fate of intestinal epithelial cells [15], we examined the expression of Notch signalling at the protein level and the change in Hes-1 and ATOH-1 at the mRNA level. Western blot analysis showed that compared with the blank group rats, IBS-D model rats exhibited significantly upregulated expression of NICD, p-ERK, and ERK and an increased p-ERK/ERK ratio. After treatment with TXYF and rifaximin, NICD and p-ERK levels and the ratio of p-ERK/ERK decreased significantly in the TXYF and rifaximin groups when compared to those in the model group (*P* < 0.05), with no significant difference between the TXYF and rifaximin groups (Fig. 4a and b). The qRT-PCR results showed that Hes-1 levels decreased while ATOH1 levels increased significantly in the TXYF group compared with those in the model group, as they did in the rifaximin group (*P* < 0.05) (Fig. 4c and d). These results suggest that TXYF-enhanced intestinal mucosal barrier function may involve the Notch signalling pathway.

### TXYF inhibited NF-κB activation in IBS-D rats

**Fig. 5:**
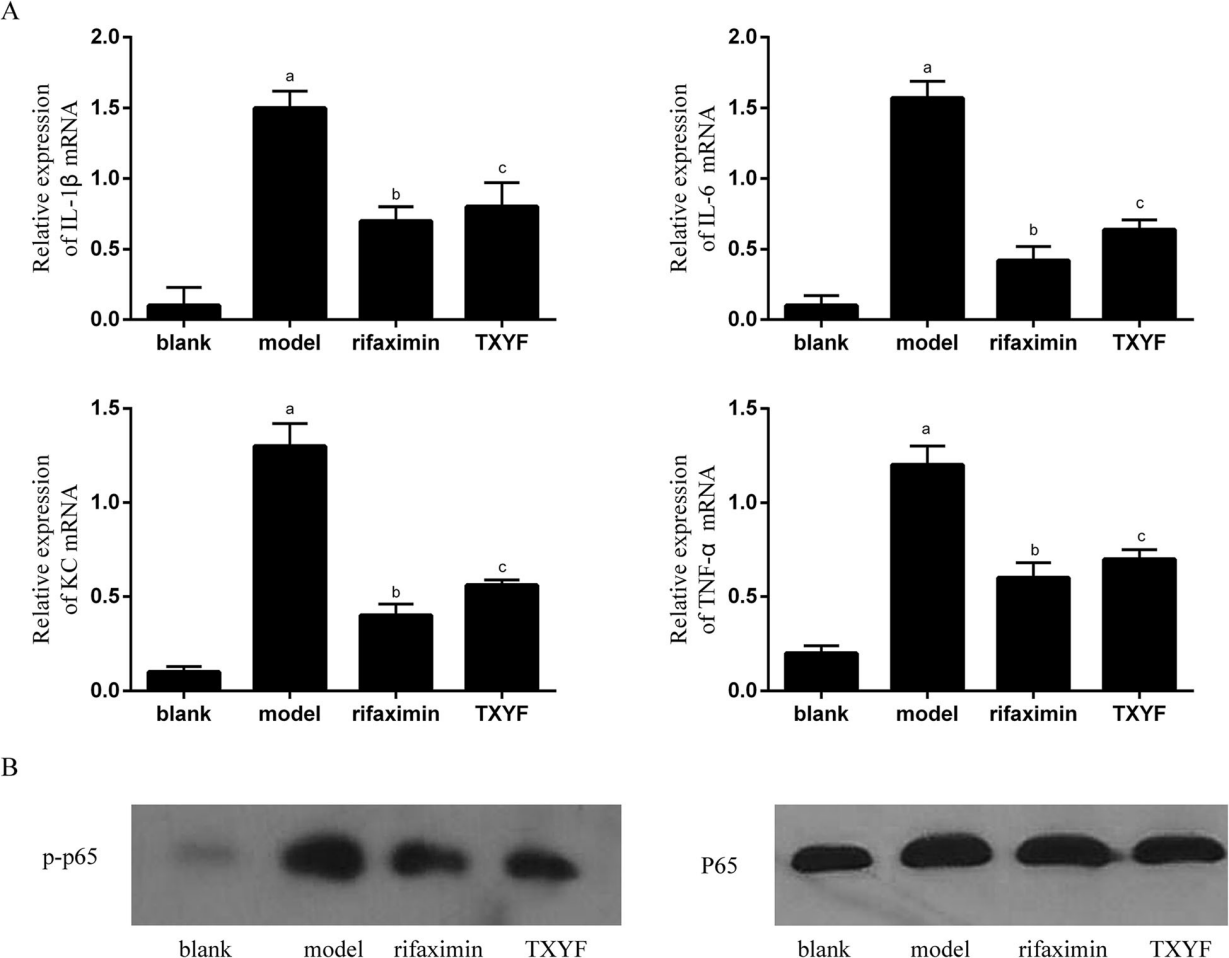
TXYF inhibits the activation of NF-κB and regulates the production of pro-inflammatory cytokines in colonic tissues of IBS-D rats. **a** qRT-PCR detection for IL-1β, IL-6, TNF-α and KC in colonic tissues. **b** Western blot analysis for p-p65 and P65 in colonic tissues. ^a^*P* < 0.05 vs the blank group. ^b^*P* < 0.05 vs the model group. ^c^*P* > 0.05 vs the rifaximin group

NF-κB acts as a major regulator of the innate immune response, which regulates the production of pro-inflammatory cytokines [16]. We used qRT-PCR to compare the mRNA expression levels of key inflammatory cytokines, such as TNF-α, IL-1β, and IL-6, and the chemokine KC. TNF-α, IL-1β, IL-6 and KC levels were significantly increased in the IBS-D model group rats compared with those in normal rats (*P* < 0.05). Compared with the model group, the TXYF and rifaximin groups exhibited significantly decreased expression of TNF-α, IL-1β, IL-6 and KC (*P* < 0.05) (Fig. 5a). Western blot analysis showed that the p-P65 level was significantly decreased in the TXYF and rifaximin groups compared with that in the model group (*P* < 0.05), while the P65 level showed no significant difference between the groups. These data strongly suggested that TXYF may indirectly decrease intestinal permeability via inhibition of pro-inflammatory cytokine production (Fig. 5b).

## Discussion

In recent years, an increasing amount of relevant research on IBS-D has been reported; however, the exact pathological mechanism of IBS-D is still unclear, and psychological and stress factors are considered to be important factors affecting IBS-D [17, 18]. In addition, an increasing number of scholars have recognized that low-grade inflammation plays an important role in secondary IBS-D [19]. As a natural physical barrier, colonic epithelial cells (CECs) play an important protective role in preventing inflammatory cells, bacteria, pathogens and other antigens from invading the intestinal mucosa [20]. The integrity of the gut barrier stabilizes the entire gut ecosystem [21]. Tight junction proteins include transmembrane proteins (such as OCLN), peripheral membrane proteins (such as ZO-1), and regulatory molecules [22]. Defects in CECs leading to the destruction of intestinal mucosal barrier function have been shown to be important pathogenic factors for IBS-D [20, 23]. Hence, restoring intestinal mucosal barrier function can be a new target for the treatment of IBS-D.

TXYF, which consists of *Rhizoma Atractylodis Macrocephalae*, *Radix Paeoniae Alba*, *Pericarpium Citri Reticulatae* and *Radix Saposhnikoviae*, is one of the most famous Chinese medicine formulas in China. It has been used in the clinical treatment of gastrointestinal diseases for more than 600 years [7]. It can effectively relieve abdominal pain and diarrhoea and restore the homeostasis of the patient’s digestive tract [8]. Twenty-three components in TXYF have been successfully verified, including monoterpene glucosides, chromones, lactones, organic acids flavonoids and steroidals, etc. [24]. However, studies on the pharmacological effects and mechanisms of TXYF in the treatment of IBS-D are still limited. Therefore, in this study, we aimed to explore the therapeutic mechanism of TXYF in an IBS-D rat model and further study the protective effect and mechanism of TXYF on the mucosal barrier. In this study, the IBS-D rat model was associated with sensitivity-related symptoms of recovery from intestinal mucosal inflammation, accompanied by high FWC and high defecation frequency. These symptoms are in accord with Chinese symptoms ‘liver qi stagnation and spleen deficiency’. After treatment with TXYF, IBS-D rats had lower FWC and higher CRD and also a decreased frequency of bowel movements, suggesting a significant decrease in intestinal permeability.

Acetic acid has long been used as a potent inducer to simulate an experimental post-inflammation recovery model to study the pathophysiological mechanisms of IBS-D and to study the mechanism and efficacy of drugs [25]. Our recent study found that intestinal permeability plays an important role in IBS-D and that miR-144 is a key regulator of intestinal epithelial barrier function in IBS-D [4]. In this study, we monitored intestinal mucosal permeability using this IBS-D rat model. We found that intestinal epithelial barrier function consists mainly of two parts: the mechanical barrier and the mucus layer barrier formed by tight junction proteins between CECs. The mucus layer consists mainly of mucin, an immunologically active substance, secreted by goblet cells and other mature intestinal epithelial cells, which can sequester the pathogenic factors in the outermost mucus and discharge them as the mucus layer into the intestinal lumen [26]. Our results showed that the intestinal mucosal barrier integrity of mucosal layers was repaired after oral administration of TXYF. Moreover, the most important finding was that TXYF could upregulate the expression of OCLN and ZO1 in the colon of IBS-D rats. These findings suggest that TXYF could effectively reduce intestinal permeability and promote intestinal epithelial barrier function in IBS-D rats.

It has been reported that the proliferation and differentiation of CECs are regulated by a variety of signalling pathways, and Notch signalling is one of them [15]. An activated fragment of Notch (NICD) activates transcriptional regulatory factors in the nucleus of epithelial cells to induce intestinal differentiation [15]. Studies have shown that abnormal expression of the Notch signalling pathway promotes the expression of the transcription factor Hes-1 while inhibiting the expression of ATOH1, leading to inhibition of intestinal epithelial cell differentiation into goblet cells, thereby weakening the mucus barrier [27–29]. Thus, activation of Notch was observed in the intestinal mucosa of the IBS-D rat model, while inhibition was observed after TXYF administration. Our data indicate that TXYF can protect intestinal epithelial barrier function by inhibiting the Notch signalling pathway.

Studies have shown that NF-κB is a nuclear transcription factor whose signalling pathway is involved in many physiological processes, including the regulation of inflammatory responses and innate immunity [30, 31]. Abnormal activation of the NF-κB signalling pathway in the mucosal layer promotes the expression of inflammatory factors, such as IL-1β, IL-6 and TNF-α [16]. Induction of the inflammatory cascade and massive neutrophil aggregation can trigger a range of pathological damage changes, such as intestinal epithelial cell damage, crypt abscesses and small-vessel vasculitis [32], which lead to an increase in mucosal immune cells and chemokines, followed by an increase in intestinal permeability. In this study, qRT-PCR showed that pro-inflammatory cytokine levels were significantly elevated in the IBS-D rat model but significantly decreased after TXYF treatment. In addition, the mucosal inflammation of the IBS-D model group rats increased, causing destruction of CECs, impairing intestinal mucosal barrier function and increasing intestinal permeability. Treatment with TXYF can inhibit NF-κB signalling, thereby regulating intestinal permeability.

## Conclusion

Taken together, our results suggest that TXYF can improve the mucosal inflammatory response during IBS-D, upregulate the expression of OCLN and ZO-1 in colonic tissues, and promote intestinal mucosal barrier function, possibly by inhibiting the NF-κB and Notch signalling pathways to repair intestinal mucosal damage. These findings confirmed that TXYF is an effective therapeutic strategy for the treatment of IBS-D, and its possible mechanisms were related to inhibiting intestinal permeability and restoring intestinal mucosal barrier function. However, in vivo studies are needed to further explore the exact protective mechanism of TXYF for intestinal mucosal barrier function.

## Data Availability

The data used to support the findings of this study are available from the corresponding author upon request.

## Abbreviations

cDNA: Complementary DNA
CECs: Colonic epithelial cells
CRD: Colorectal distension
ELISAs: Enzyme-linked immunosorbent assays
FWC: Faecal water content
HE: Haematoxylin-eosin
IBS-D: Diarrhoea-predominant irritable bowel syndrome
ig: Intragastric administration
OD: Optical density
SD: Standard deviation
TXYF: Tong-Xie-Yao-Fang
